# Notes and News

**Published:** 1895-06-29

**Authors:** 


### Notes and News.

(Collected and Communicated.)

Dr. Thorne Thorne has been elected a foreign member of the Société Française d'Hygiène.

The Metropolitan Hospital Sunday Fund collection has now reached £30,000.

Dr. James Reid, C.B., resident medical attendant to the Queen, has been appointed a Knight Commander of the Bath.

The Prince and Princess of Wales have consented to attend at the formal opening of the new front of the Royal Free Hospital.

The manly and eloquent sermon preached by Archdeacon Sinclair in St. Paul's Cathedral on Hospital Sunday is being published, at request, by the Scientific Press.

There is a growing feeling that to the habitual consumption of absinthe is due half the moral and physical vils from which France, and especially its urban population, suffer.

A school for deaf-mutes is being erected at Athens, and will, it is expected, be ready for occupation in September. A payment will be required until voluntary contributions permit of free admission. According to official returns, there are 1,100 deaf mutes in Greece.

We have frequently pointed out that other methods more attractive than the annual meeting, and less expensive than the festival dinner, might be adopted with advantage to hospitals to gather together their governors and others whom it is desirable to interest. We are glad to see that the management of the Royal Free Hospital are about to try an experiment of the kind we advocate. On July 1st the chairman and others in authority will hold a conversazione, which those connected with the hospital, together with their friends and others interested in the work of the hospital, are invited to attend.

The Prince of Wales will lay the foundation-stone of the Lower School of the Royal Medical Benevolent College at Epsom on Tuesday, July 9th. A train will leave Victoria at two o'clock, in time for the ceremony. The event will be one of great interest, especially to those connected with the medical profession.

A new wing in connection with the Kendray Infectious Hospital has just been opened at Barnsley. The addition, costing £2,000, has been erected through a bequest from the late Mrs. Lambert, and the wards will be called after her name. The new premises are connected with the administration block by means of a covered corridor, and consist of one male and one female ward, two small special wards, and a nurses' day room. The older portion of the Kendray Hospital was also the gift of Mrs. Lambert to Barnsley.

The Australia Health Society, with the view of popularising the study of hygiene, has just held an examination of all the pupils that chose to present themselves from all the State schools within a radius of ten miles from the Melbourne Post Office. Fourteen schools accepted the invitation, 398 candidates presented themselves, and of these 170 have passed. The examination was held on the text-book used in the schools, "Manual of Health and Temperance," by Thomas Brodribb, formerly chief inspector of State schools.

The meetings of dispensary committees in Ireland are not seldom enlivened by strange and amusing proceedings. Even in Ireland, however, the fiasco which occurred recently at Cork was a little startling. Through the mistake of the clerk to the guardians two different hours for a meeting were received by the members of the Dispensary Committee. The members first to arrive transacted their business and departed. The second meeting duly assembled, and its proceedings were of a lively character. The members appeared to agree upon no point except in objecting to the decisions of the earlier meeting, and finally dispersed to obtain legal opinion upon the whole matter.

The Metropolitan Provident Medical Association held a meeting last week for the purpose of discussing the work of the association. Mr. Gerald Balfour, M.P., presided, and among those present were Lord Battersea, Canon Ainger, Sir William Mackenzie, Sir Spencer Wells, Mr. T. H. Bryant, Mr. T. Holmes, and a large number of gentlemen of the medical profession. The Chairman said the institution was not, in the ordinary sense of the term, a charitable institution, as it encouraged the people to help themselves. Lord Battersea remarked that no fewer than 30,000 people had benefited by the association, and the working classes had contributed more than £4,000 a year in periodical payments. At the close of the meeting it was resolved: "That the gratuitous and sometimes indiscriminate treatment of persons in the out-patient and casual departments of hospitals results in the creation of a pauperised spirit among the industrial classes, which evil is best met by the establishment of self-supporting provident dispensaries in co-operation with the hospitals, and that funds for promoting this object are urgently needed by the association."

